# A Novel Approach for a Chip-Sized Scanning Optical Microscope

**DOI:** 10.3390/mi12050527

**Published:** 2021-05-06

**Authors:** Joan Canals, Nil Franch, Victor Moro, Sergio Moreno, Juan Daniel Prades, Albert Romano-Rodríguez, Steffen Bornemann, Daria D. Bezshlyakh, Andreas Waag, Florian Vogelbacher, Stefan Schrittwieser, Katarzyna Kluczyk-Korch, Matthias Auf der Maur, Aldo Di Carlo, Angel Diéguez

**Affiliations:** 1 Department of Electronic and Biomedical Engineering, University of Barcelona, 08028 Barcelona, Spain; nfranch@ub.edu (N.F.); vmoro@ub.edu (V.M.); sergiomoreno@ub.edu (S.M.); dprades@ub.edu (J.D.P.); albert.romano@ub.edu (A.R.-R.); angel.dieguez@ub.edu (A.D.); 2 Institute of Semiconductor Technology, Technische Universität Braunschweig, 38106 Braunschweig, Germany; steffen.bornemann@tu-bs.de (S.B.); d.bezshlyakh@tu-bs.de (D.D.B.); a.waag@tu-braunschweig.de (A.W.); 3 Molecular Diagnostics, AIT Austrian Institute of Technology, 1210 Vienna, Austria; vogelbacher@ieee.org (F.V.); stefan.schrittwieser@ait.ac.at (S.S.); 4 Dipartimento di Ingegneria Elettronica, University of Rome Tor Vergata, 00133 Rome, Italy; katarzyna.kluczyk@uniroma2.it (K.K.-K.); matthias.aufdermaur@uniroma2.eu (M.A.d.M.); aldo.dicarlo@uniroma2.it (A.D.C.)

**Keywords:** chip-size microscope, nanoLEDs, scanning optical microscopy, lensless, shadow imaging

## Abstract

The recent advances in chip-size microscopy based on optical scanning with spatially resolved nano-illumination light sources are presented. This new straightforward technique takes advantage of the currently achieved miniaturization of LEDs in fully addressable arrays. These nano-LEDs are used to scan the sample with a resolution comparable to the LED sizes, giving rise to chip-sized scanning optical microscopes without mechanical parts or optical accessories. The operation principle and the potential of this new kind of microscope are analyzed through three different implementations of decreasing LED dimensions from 20 µm down to 200 nm.

## 1. Introduction

In the last two decades, on-chip microscopy based on computational imaging has received much attention due to its clear advantages as a low-cost biomedical research and diagnostic tool over conventional optical microscopy by providing high resolution and a large field of view (FOV) simultaneously [1]. Among the different computational microscopy implementations [2], lensless microscopy has been extensively used because of its versatility and flexibility to implement different techniques, from shadow imaging to fluorescence [3,4,5,6,7], while keeping the microscope implementation simpler. Lensless microscopy relies on the traditional microscopy principle, where the analyzed sample area is illuminated homogeneously by a single light source, and the scattered light from each point is collected by an area-selective detector providing the spatial resolution, commonly a high-resolution image sensor. Then, the captured diffracted shadow pattern is used to reconstruct the object image digitally.

The typical lensless scheme requires placing the sample away from the light source (>5 cm) to consider the illumination light as a planar wave and close to the sensor (less than 1mm) to maintain a unit magnification gain to the sensor plane, where the pitch and size of the pixels determine the resolution of the image [8]. However, the spatial resolution of lensless microscopy is reaching its limit. Pixel sizes smaller than 1 µm are challenging to achieve in CMOS technologies [9,10]. To overcome this pixelation limit, several techniques known as pixel-super-resolution have been developed, achieving resolutions below 1 µm by shifting the illumination source [8,11], reaching the diffraction limit by scanning the illumination angle across a dome surface [12]. Although all these techniques provide a high-resolution image with wide FOV, they require high computational power to reverse-engineer the diffraction patterns into images [13].

**Figure 1:**
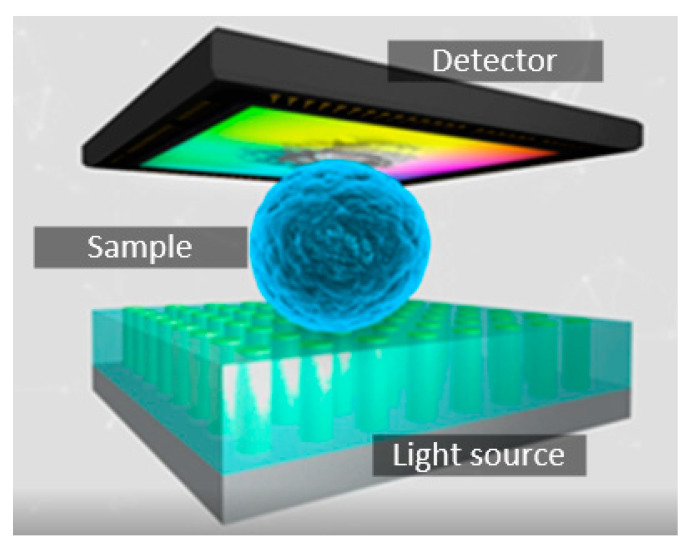
Illustration of the spatial resolved illumination-based scanning optical microscope. The specimen lying on the nanoLED chip surface is scanned, while the projected shadow intensity is recorded.

A completely new approach to conventional lensless microscopy was presented in [14], where a spatially resolved illumination source provides the microscope resolution instead of the detector system. As depicted in Figure 1, the traditional lensless setup is reversed. A structured light source, composed of homogeneously distributed tiny individually addressable elements, illuminates the sample. Whenever a single emitter is activated, the light propagation depends on the sample morphology directly above it. Therefore, to obtain an image, the light intensity transmitted through the sample region is sensed by an optical detector, activating one light element at a time and thereby scanning across the sample space. If the specimen and the light source are in close contact, the system spatial resolution is mainly determined by the emitter’s pitch. Consequently, the constraints in the detector are simplified, and an arbitrary photodetector can be used to collect the transmitted light, since the spatial resolution is given by the illumination source. Thus, the microscope size is reduced to the measurement cavity formed by the key elements: the structured illumination device and the integrated optical detector [15].

This straightforward technique generates the shadow images without any computational need, since the transmitted light intensity through the sample is mapped directly by the array of light sources. Furthermore, for light element sizes in the nanometer regime, below the diffraction limit, and with the sample in close contact with them, super-resolution imaging may become possible with a chip-based microscope following this approach [16].

In this work, the different components and microscope prototypes built to demonstrate the feasibility of this new type of microscopy are presented. First, the structured light source was achieved by an array of light-emitting diodes (LEDs). Although LEDs have conquered the market for general lighting applications [17] due to their superior characteristics compared to other traditional light systems; e.g., the halogen-based emitters, there are still no structured LED arrays with individually addressable submicron pixels commercially available. Thus, sub-µm LED arrays were developed [18] to build up the first microscope prototypes and validate this new technique. On the other hand, high-sensitive light detectors are required to detect the light emitted by the submicron LEDs. Several CMOS cameras, including CMOS single-photon avalanche diode (SPAD), were used in the prototypes. Although in this work, we present prototypes working in the blue range of the visible spectrum, this new microscopy method will work independently of the wavelength of the light being emitted, as long as the light source and the sensor are compatible.

In particular, we focused on the implementation of three microscope prototypes, each based on unique LED arrays with different pixel sizes, pitch, and array elements, demonstrating the potential to implement a true chip-sized scanning optical microscope without moving parts. In the following subsections, we describe the implementation and characteristics of the different microscopes’ setups and their operation principles to finally present their major results.

## 2. Materials and Methods

### 2.1. Microscope Architecture Overview

**Figure 2:**
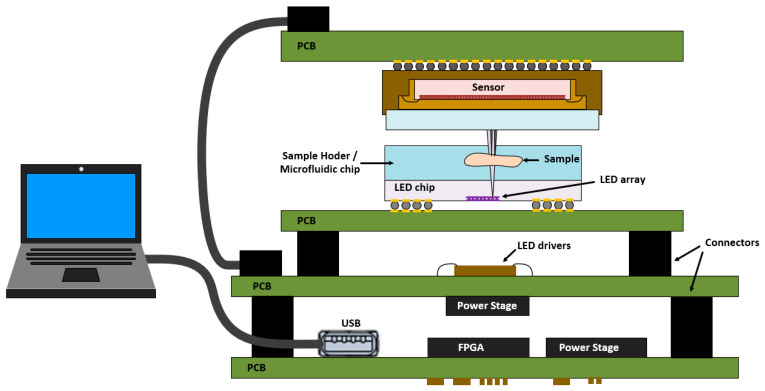
Scheme diagram of the basic stack-up structure used in all the microscopes.

As mentioned above, the three microscope setups were assembled based on three different LED array configurations to explore the technology potential progressively. Through all the microscope versions, the system architecture was maintained, since the microscope principle is the same for all of them. Figure 2 shows the basic microscope stack-up structure implemented, with the sample laying over the LED array chip and the optical sensor on top collecting the transmitted light. The architecture was designed modularly to provide flexibility to test each microscope element independently and allow easy replacement without compromising the rest of the system. To that end, each component of the microscopes was implemented on a separated printed circuit board (PCB) carrier. All the key elements of the setup (LEDs, the drivers, and the CMOS sensor) were connected to an FPGA board. The FPGA controls the microscope operation, with all the routines such as image acquisition, calibration, and test procedures implemented at the hardware level, relegating the computer to displaying images and selecting configuration settings through the graphical user interface.

#### 2.1.1. LED Chips

Three different planar GaN-based blue LED array architectures using direct and matrix addressing were constructed. With direct addressing, each LED of the array is individually driven through a specific contact line, requiring N × M connections for an N × M size array. In contrast, in matrix addressing, each LED is selected through its row and column contact lines, reducing the number of control signals to N + M for the same array. The emission peak for all the LED chips was centered at 450 nm.

**Figure 3:**
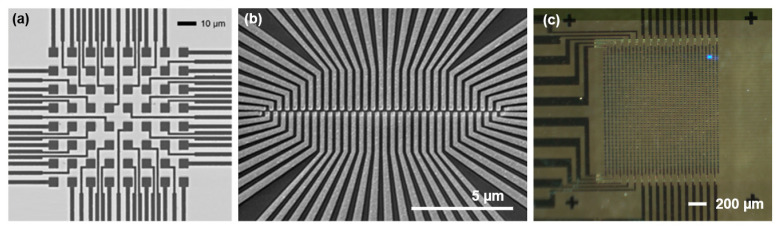
(**a**) Led1: direct addressing LED array chip with 8 × 8 5 µm pixels regularly spaced at 5 µm. (**b**) Led2: 2 × 32 direct-addressing linear array LED chip with pixels sized 200 nm. (**c**) Led3: 32 × 32 matrix-addressing LED array of 20 µm pixel size and pitch.

The first LED array, named Led1, implemented a direct-addressing approach in an 8 × 8 array. The LEDs had a square shape with 5 µm sides, regularly spaced with a 10 µm pitch (Figure 3a). The final chip had 8 n-contact pads running as a common n-GaN contact and 64 p-contacts surrounding the LED array, and presented a cut size of 1 cm × 1 cm. The fabrication process was the metal-oxide-GaN (MOGaN) process reported in [17], which employed an insulating layer of SiO_2_, which was opened up via photolithography and etching steps to define the p- and n-contact areas. The metal stacks of the n- and p-contacts were optimized to ensure proper ohmic contact. Finally, each gold-terminated pixel was connected to one p-contact pad via a gold lead. Since the Led1 presented a land grid array (LGA) contact pattern, it was assembled to the carrier PCB using a low-temperature bonding process based on standard industry stencil printing [19], but using a silver-based conductive epoxy (CW2400 from Chemtronics Circuit Works, Kennesaw, GA, USA) instead of solder paste. The procedure adopted avoided the stress generated on the contact pads by the different expansion rates of the sapphire and the PCB, produced in a standard flip-chip reflow soldering [20], which may damage the contact pad.

The second LED chip, Led2, consisted of 2 × 32 direct addressable 200 nm nanoLEDs with a 400 nm pitch. The array configuration illustrated in Figure 3b presented a shift of 200 nm in the alignment of the columns of the different rows to give an effective pitch of 200 nm when a sample crossed perpendicular to the array. The final chip size was 7.1 × 8.5 mm with 64 p-pads and 4 n-pads located on the chip’s sides, at a distance of 6 mm. The fabrication process was the same as that of Led1, but using electron beam lithography (EBL) instead of photolithography, which was necessary to achieve submicron LED structures as detailed in [21].

The third LED chip (Led3) followed a completely different architecture. It consisted of 32 × 32 matrix-addressable 20 µm LEDs spaced 20 µm. The fabrication process relied on deep-etching parallel fins into a GaN-wafer down to the underlying sapphire, ensuring electrical insulation between the fins, which functioned as n-contact lines [22]. To do so, a Cr hard mask was deposited to define the fin structure. Next, the n-contact openings were defined at the ends of the fins by an additional etching step. Before applying orthogonal metal lines on top of the fins, the space between the fins was filled with benzocyclobutene (BCB), which acted as an insulating polymer. A mechanical polishing was performed down to the Cr mask to ensure good planarity of the surface. After the planarization step, the chip surface was insulated with an SU-8 with openings directly over the array area and at the larger end of the fins, where n-contact pads were subsequently created by etching the BCB down to the n-GaN and applying Ti/Au metal stack. Finally, orthogonally running p-contact lines were done by the usual lift-off process, applying Pd and Au for ohmic p-contacts (a semitransparent metal stack), and a final insulating SU-8 layer to protect the metal contact lines. The Led3 chip (Figure 3c) was designed to have the same configuration as the Led2 chip, the same dimensions, and pad layout, placing all the p-contacts on the right side and the n-contacts on the left.

#### 2.1.2. LED Array Driver

**Figure 4:**
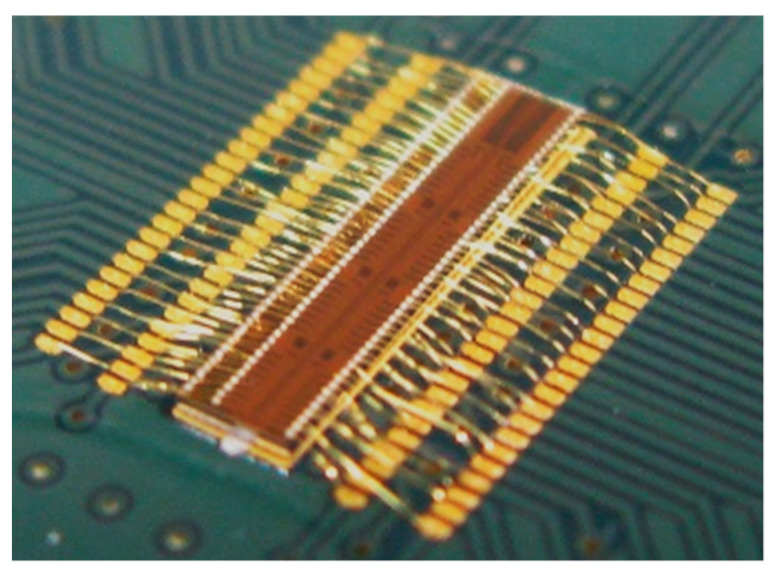
Integrated HV-CMOS LED driver chip, wire-bonded in a matrix-addressing configuration.

In order to control the different LED arrays, a specific driver chip (from here on, called the driver) was fabricated in a 0.35 µm HV-CMOS process. The driver consisted of 64 anode and 32 cathode driving circuits arranged in a single chip of 1.76 mm × 7.32 mm (Figure 4). The driving circuits were distributed in three rows across the chip, where the outer ones were the anode drivers and the central row the cathode. Thus, the driver chip could manage up to 64 direct addressable or 32 × 32 pixels with a matrix-addressing scheme. Both drivers could generate pulses (from an external trigger signal) with selectable amplitude from 3.3 V up to 10 V and widths down to 700 ps and 10 ns at full width half maximum (FWHM) for the anode and cathode driver, respectively. The cathode driver was designed to maintain a positive voltage (between 3.3 V to 10 V) to the unselected LEDs, preventing them from turning on while generating a reverse pulse (from bias voltage to 0 V) on the selected LED.

#### 2.1.3. Optical Detector

**Figure 5:**
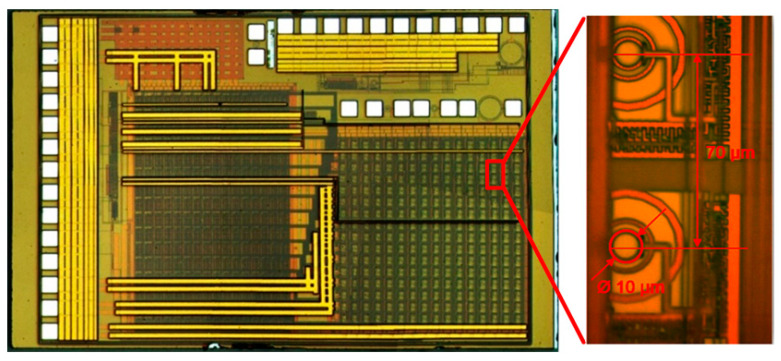
CMOS SPAD camera with a detail of the 10 µm circular pixels.

As a further component, a CMOS SPAD camera was designed in a 0.35 µm HV-CMOS process. The camera was composed of an array of 16 × 16 pixels, with each pixel including the 10 µm diameter sensor, the readout, and control electronics. Each pixel’s output was connected to a dedicated 8-bit counter per pixel and bridged directly to one common output for all the pixels. The designed pixel presented a dark noise of 200 Hz and a photo-detection probability of 10% at 450 nm [23]. The low profile provided by the bare die directly wire-bonded on a custom PCB (Figure 5) allowed us to place the image sensor as close as possible to the sample.

Alternatively, a commercial CMOS image sensor was also used as a light sensor due to the advantages it provided: a bigger FOV for searching the area of interest, relaxation of the system alignment, and the possibility to define the sensing area size and position. The used sensor was the Aptina MT9V024 camera module (from OnSemiconductor®, Phoenix, AZ, USA). The sensor has a monochromatic array of 744 × 488 pixels of 6 µm with an 8-bit dynamic range, resulting in a 4.55 mm × 2.97 mm sensing area. The pixel array implemented a global shutter that could provide 76 fps at full resolution. The sensor was mounted on a commercial board (DMM-22BU, C03-ML, from The Imaging Source Europe GmbH, Bremen, Germany) and was controlled through a USB interface.

### 2.2. Microscope Operation

#### 2.2.1. Transmission Image Reconstruction

**Figure 6:**
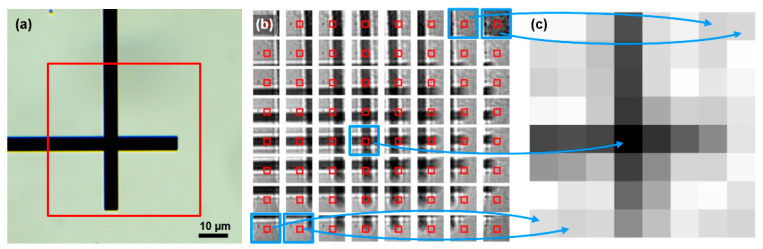
Transmission image reconstruction of the intersection of two lines of 5 µm width using the Led1 and MT9V024 image sensor as a light collector. The sensing area used was 9 × 9 pixels. (**a**) Optical image of the intersection of two lines of 5 µm width with the observed area highlighted in red. (**b**) Composition of the different raw shadow images generated by each micro-LED with the sensing area highlighted in red. (**c**) Reconstructed transmission image using the spatially resolved illumination.

The transmission images of the sample region directly over the LED array were generated as follows. The LEDs were sequentially switched on and off, scanning the sample. One frame of the image sensor was acquired per each LED. The same sensing area of N × N pixels of each frame was selected to measure the total intensity emitted per LED through the sample. Finally, the measured intensities were arranged to create an N × M (the size of the LED array) transmission image that offered information about the shape of the object under investigation at these particular LEDs-on positions. Figure 6 illustrates the transmission image reconstruction using Led1 and the MT9V024 CMOS image sensor. Figure 6b shows the composition of raw lensless shadow images of the structure (Figure 6a) under study generated by each LED (with the common sensing area highlighted in red), and Figure 6c shows the reconstructed transmission image by integrating the intensity in the sensing area (9 × 9 pixels) for each LED and arranging them according to their position within the LED array.

The quality of the reconstructed image in terms of contrast is limited by the relationship of the distances between the sensor, the sample, and the light sources, as well as the size of the detection area, as shown in [20], where this technique was reported for the first time. To produce sharper contrasted images, the sample should be placed as close as possible to the light sources, and the size of the observed objects must be larger than twice the LED pitch according to sampling theory. Otherwise, poor contrasted images are obtained, which could present aliasing in a limit case.

#### 2.2.2. LED Array Equalization

Since the emission of the LEDs in a single array can vary by more than 30%, it is essential to equalize them for correct image reconstruction. For this purpose, each LED emission was dimmed by pulse width modulation (PWM) to a user-defined target intensity, reducing the inhomogeneity in the array emission below 2%. The PWM period must be higher than the measurement window of the photodetector used (the exposure time in the CMOS sensors) to avoid detecting light modulation. First, the camera was fixed over the LED array in the distances necessary for the measurement, but without a sample inserted. Next, the sensing area (size and position) and the target intensity were selected, taking care to avoid saturating the sensor and using a small sensing area centered with the LED array, since they had a direct impact on the contrast of the reconstructed image [24]. Once the camera position and all user-defined parameters were set, each LED was individually turned on, and its intensity was measured and equalized by varying the duty cycle of the PWM driving signal until it matched the target intensity. The resulting duty cycles associated with each LED were stored in a look-up-table for later use during image acquisition.

### 2.3. Test Sample

As is often the case in scanning microscopy techniques, the areas studied were mainly the surfaces of samples. Thick objects can also be partially observed, but with less resolution because the information from thick objects is lost as soon as the light is absorbed within the sample. Given this, and that the structure of the scanning LED array was fixed on the plane, this microscopy method was suited only for samples as flat and thin as possible. To validate the prototypes, a set of patterns (with structures from 50 nm up to 20 µm) fabricated by aluminum EBL was used to characterize the resolution of the microscopes.

The EBL sample fabrication was performed on device-quality 4” fused silica wafers (0.525 mm thick). First, the wafer was dehydrated in an oven at 250 °C for 2 h. Next, a nominally 180 nm-thick CSAR-P6200-09 positive photoresist was spun at 4000 rpm for 1 min and cured at 180 °C for 3 min. Before the exposure with a beam current of 2 nA, a 20 nm-thick aluminum layer was thermally evaporated at 0.3 nm/s to reduce charge build-up in the wafers. The aluminum layer was removed by a 60” single bath in a 2.38% tetramethyl–ammonium hydroxide solution, and the development of the CSAR photoresist was done using the developer AR 600-546 for 1 min. The sample was further covered by a 40 nm-thick electron-beam evaporated chromium layer, deposited at 0.5 nm/s. Finally, the lift-off of the deposited metal was achieved in Remover 1165 at 45 °C for 20 min, followed by rinsing in isopropyl alcohol.

## 3. Results

### 3.1. First Microscope Generation

**Figure 7:**
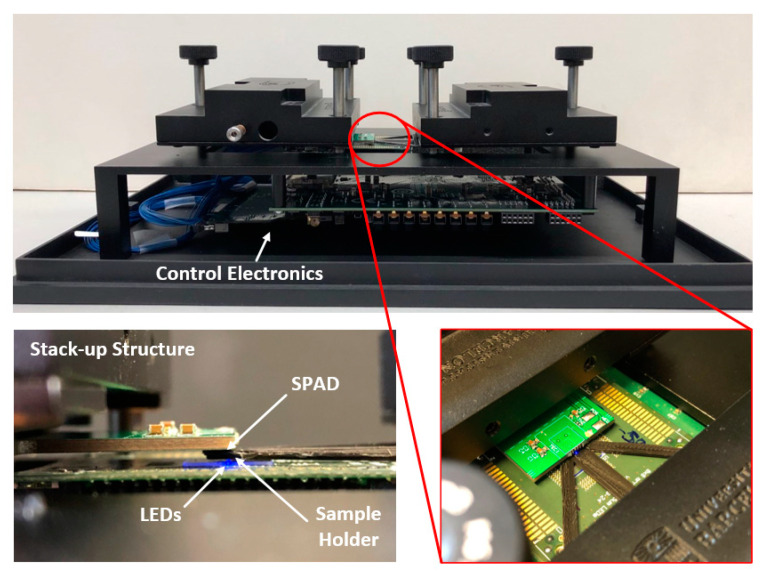
Setup of the first-generation microscope with a detail of the stack-up composed by the Led1 chip, sample/sample holder, and CMOS SPAD camera.

The first microscope was based on the Led1 chip and the CMOS SPAD camera. For this implementation, the Led1 chip was fixed at the bottom, whereas the sample rested on the sapphire substrate with two (XY) degrees of freedom. The camera was positioned on top of the stack-up by means of the XYZ microstager. A custom 3D-printed sample holder was designed to move the sample in direct contact with the Led1 chip. The rest of the setup was fabricated, including the two microstages used to align the sample and the camera with the LED chip, by a 3D aluminum computer numeric control (CNC) machining. The setup was enclosed in a dark box measuring 31 × 21 × 12 cm^3^ (Figure 7).

A complete analysis and characterization of this microscope were reported in [22]. N. Franch et al. validated the technique, demonstrating that the principle of the microscope relied on the close contact of the sample under study with the LED array, providing a spatial resolution (the ability to identify two nearby objects) of two times the LEDs’ pitch, and a FOV determined by the size of the LED array used.

As in scanning microscopy techniques, the time to construct an entire image depends on the number of scanning steps (64, total LEDs of the array) and the time each step takes. For this first prototype, the scanning speed was 1000 LED/s (or samples per second).

**Figure 8:**
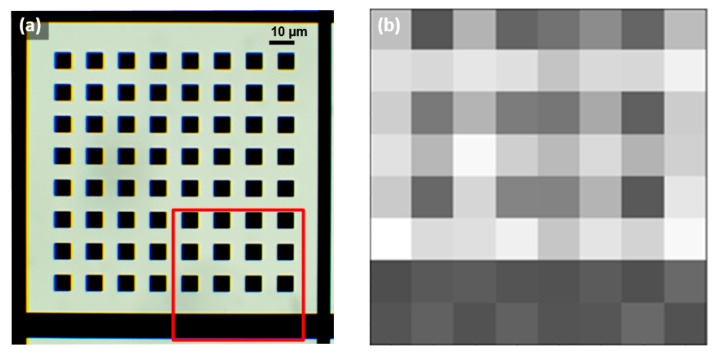
(**a**) Optical image of the EBL pattern of 6.4 µm squares regular spaced at 6.4 µm, with the observed region highlighted in red. (**b**) Image reconstructed with the microscope prototype based on the 8 × 8 5 µm LED array chip (Led1), which presented aliasing, indicating the limit to observe periodic objects of sizes comparable to the LED.

Figure 8 shows the smallest resolved patterns observed (an array of 6.4 µm squares) with this microscope setup. The poor quality of the reconstructed image compared to the optical one was, according to sampling theory, because the size and periodicity of the squares were similar to the size and pitch of the LEDs (6.4 µm/12.8 µm and 5 µm/10 µm, respectively). Therefore, the 6.4 µm squares were not imaged correctly in the central area because they were aliased. Additionally, the FOV for this setup was smaller than the LED array size because the microscope was operated in far-field conditions since the emission of the LEDs was through the sapphire substrate. This set a vertical distance between the sample and the LED array of 300 µm, which, conjointly with the sample-sensor distance (~600 µm) and the sensing area used (a single 10-µm SPAD detector), reduced the FOV from 75 × 75 µm^2^ down to 53.4 × 53.4 µm^2^.

### 3.2. Second Microscope Generation

**Figure 9:**
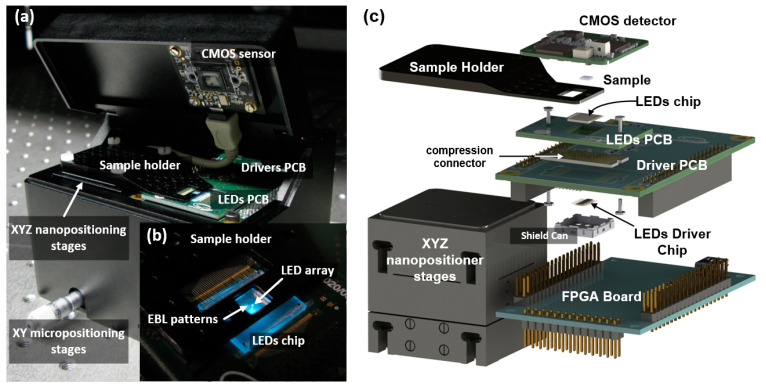
(**a**) Image of the microscope configuration with nanopositioner stages to extend the FOV, and (**b**) a detail of the sample over the LED array. (**c**) Exploded view of the second generation microscope stack-up structure with nano-stages.

The second microscope generation was based on the Led2 chip. Unlike Led1, the Led2 chip configuration allowed the emission through the p-contact metal lines, reducing the critical distance (emitter-sample) to the minimum. Another problem faced with the first generation was the limited FOV of the array, which in this case was critical due to the smaller pixel size (200 nm) and the array configuration of Led2 (2 rows of 32 elements). However, the Led2 chip was designed as a high-resolution scanning line array, which required moving the sample orthogonally over it, thus extending the FOV. Therefore, a custom sample holder attached to a nanopositioning system was fabricated (by 3D aluminum CNC machining) to move and hold the sample in direct contact with the LEDs inside the observation cavity. The nanopositioning system was formed by a compact XY piezo stage (P-621.2CD, from Physik Instrumente GmbH & Co. KG, Karlsruhe, Germany) stacked on a vertical Z piezo stage (P-621.2ZCD, from Physik Instrumente). Both actuators presented a 0.1 nm resolution and 100 µm travel range, with a positioning accuracy of 0.02%. To extend the movement range for coarse position of the sample, XY micropositioning stages were integrated into the microscope shield (Figure 9a).

The speed of this second microscope was only 20 LEDs/s. Nevertheless, this setup was not aimed at maximizing the scanning speed, but to study the resolution improvement using smaller LEDs.

As shown in Figure 9c, the implementation presented the basic stack-up structure of the first generation. However, in this setup, the Led2 chip was placed in a recess on the PCB (providing a flat surface with the PCB for the sample holder) and wire bonded to it (Figure 9b). The connection to the LED driver PCB was made through an ultra-low-profile compression connector (ZA1-20-2-1.00-Z-10 from SAMTEC Inc., New Albany, IN, USA), simplifying the LED carrier board to its bare minimum without any other components (not even a connector). At the bottom, the LED drivers were wire-bonded and protected with a shield cap. The entire setup was enclosed in a custom aluminum CNC-machined case (measuring 11 × 8.7 × 7.4 cm^3^) that provided the dark environment required for the measurement. The CMOS sensor used (the MT9V024) was attached to the hatch, allowing direct access to the LED chip and the sample when open, and placing the sensor at 1.9 mm from the LED surface when closed.

**Figure 10:**
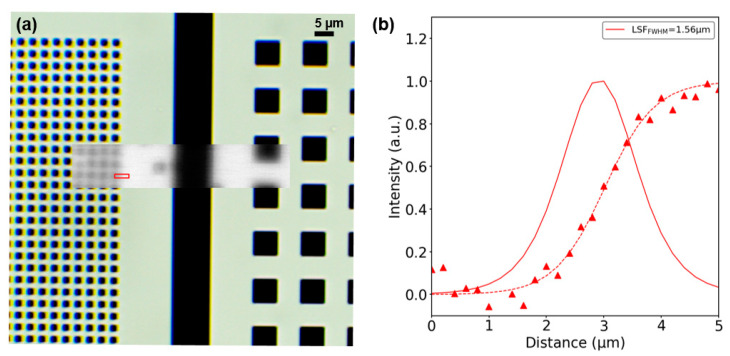
(**a**) Optical image of the 1.6 µm (left) and 6.4 µm (right) squares of the EBL pattern, with the reconstructed image of the same region superposed. (**b**) The ESF and the LSF calculated on the specified region of the inset image.

With this setup, smaller EBL structures were observed (Figure 10a). However, due to the performance of the LED array in which only a few LEDs worked, we decided to use only one LED and perform a 2D scan by moving the sample, emulating a larger LED array. Figure 10a shows the reconstructed image of the EBL pattern region with 1.6 µm and 6.4 µm squares, using a step size of 200 nm in both directions. The spatial resolution was determined by the edge spread function (ESF) and line spread function (LSF) methods [25] measured between 10–90%. The extracted resolution from one of the 1.6 µm square’s edges was 1.56 µm (Figure 10b), four times the expected resolution (400 nm), since the sample was placed directly over the nanoLEDs. However, further analysis of the nanoLED array configuration (by finite difference time domain simulations [26]) showed that the metal structure and the depth of the light emitter region affected the spot shape and size at the chip surface, degrading the resolving power of the setup.

The poor reconstruction of the 1.6 µm squares in Figure 10 was because the size of the light spot was comparable to the observed squares and their periodicity, thus reducing the contrast of the image in this region, even when scanning with a step eight times smaller (200 nm). This showed that the image quality (in terms of contrast and resolution) did not depend only on the LED pitch, but also on the light spot size on the sample plane.

### 3.3. Third Microscope Generation

The third microscope generation was not designed to improve the resolving power of the microscope. Instead, the intent was to study a matrix-addressing connection, thanks to which the FOV was much larger with the same number of connections, taking aside the size of the pixel, since each pixel was addressed by its column and row contact lines. A total of 1024 pixels arranged in a 32 × 32 array were addressed using only 64 driving circuits. Thus, the control electronics for large arrays were simplified while solving the scalability problem presented by the direct-addressing approach. Furthermore, this implementation shows the simplicity of measurement and easy sample positioning for large arrays.

The microscope was implemented using the matrix-addressing Led3 chip. Since the Led2 and Led3 chips were designed to have compatible configurations (die size and contact pads layout), the Led3 chip was compatible with the second-generation microscope setup, with two minor changes. First, the LED driver connection was changed because the matrix scheme required 32 cathode drivers and 32 anode drivers, and second, a minor upgrade of the FPGA firmware was implemented according to the new driving scheme.

**Figure 11:**
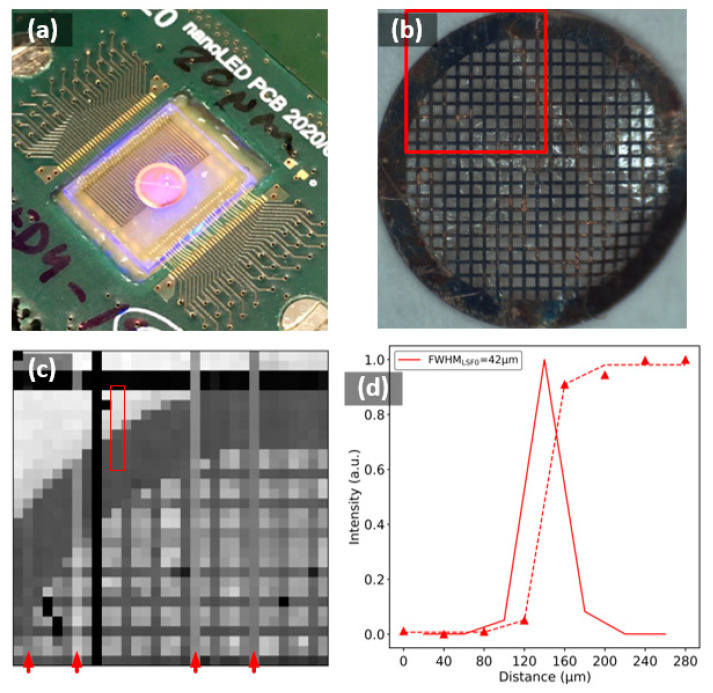
(**a**) EM-Tec TEM over the Led3 chip. (**b**) EM-Tec TEM square mesh support grids, 200 mesh, 90 μm hole, 37 μm bar with the observed area highlighted. (**c**) Reconstructed ChipScope image of the highlighted region with 20 µm Led3 version. The red arrows indicated the vertical artifact lines due to emissions coming from some p-contact lines. (**d**) The ESF and the LSF calculated on the specified region of the image in (**c**).

Figure 11 shows a reconstructed image of an EM-Tec TEM support grid (200 mesh with 90 µm holes and 37 µm bars) placed directly over the LED array without any holder. The scanning speed was 60 LEDs per second, determined by the frame rate of the camera used. The reconstructed image presented some dead pixels (black ones) and vertical artifact lines due to emissions from some p-contact lines (identified by red arrows in Figure 11c), directly affecting the image quality in contrast and reconstruction. This reflected how, in the matrix-addressing approach, a failure in a contact line had a high impact on image reconstruction, since a whole line of the LEDs was affected, as opposed to direct addressing, where only one LED/pixel from the resulting image was affected. Even so, the edge of the grid and the mesh can be identified on the top left side of Figure 11c. However, the TEM grid used was at the limit of the resolution of this microscope (based on Led3: 32 × 32 20 µm LED array with 40 µm pitch) according to the previous results. The spatial resolution extracted by the ESF and LSF methods measured between 10–90% was 42 µm (Figure 11d), showing that in this case, the pitch of the LEDs was the limiting factor instead of the size of the light spot. Therefore, to resolve two objects, they must be spaced at at least twice the pitch of the LEDs. As shown in Figure 11c, the mesh bars were not correctly sampled, and not all spaces between bars were sampled equally, affecting the contrast of the image, which was already poor because of the defective pixels on the LED array.

## 4. Discussion

In summary, we have presented a new approach toward on-chip scanning optical microscopy, based on the miniaturization of light sources instead of the sensor geometry. The novelty of this technique relies on the use of individually addressable nanoLEDs to scan the sample in close contact to provide a direct mapping of the sample without requiring focusing systems or mobile parts, and just measuring the received intensity. The presented devices allow us to observe samples unresolvable by plain human sight, offering information about the structure observed. The resolving power of microscopes constructed using this technique is set, in accordance with sampling theory, by the distribution of the LEDs, and for the microscopes constructed so far, this went from being able to resolve 20 µm objects in the first prototype down to 3.2 µm with the second generation.

Since the resolving power relies on the light sources, the proposed technique relaxes the requirements on the sensor side, where an arbitrary detector can be used. Nevertheless, a multiple pixel sensor provides significant advantages over using a single one as in [22], such as sample previsualization, variable sensing, and multiple sensing areas. Furthermore, the straightforward implementation of this technique without image postprocessing makes it ideal for integration, enabling a true low-cost chip-size microscope, as opposed to other microscope techniques based on the transport of intensity equation [27], such as ptychography [7] or digital holographic microscopy and its variants [28,29,30], which combine wide FOV with submicron resolution, and even 3D reconstruction, which requires complex optical setups and high computational power to recover the image.

The operation principle was validated using three different LED array configurations, showing that the system resolution was primarily determined by the LEDs’ size and pitch, with the sample placed in contact with the emitters. Although the presented prototypes had a small FOV, and the submicron resolution was not achieved even with 200 nm LED arrays, there is still room for improvement by adopting different strategies; i.e., using transparent conductive oxides for the contact lines, thinning the insulating layer between the metal pattern and the LED structure, and of course, reducing the LED size.

However, the future of this new kind of microscope relies on the matrix-addressing approach thanks to its scalable nature. Matrix addressable arrays have the potential to create large arrays of thousands of nanoLEDs. Compared to current microdisplay technology that implements a hybrid interconnection to solve the routing problem of direct addressing [31], matrix addressing performs even better because it leverages the limit of the CMOS backplane circuits as well [25]. With matrix addressing, the number of connections is minimized, simplifying the interconnection scheme and, consequently, driving electronics.

While a large FOV is desirable, it increases scanning time by the same proportion, a problem that should be tackled. Since LEDs have been shown to be able to switch at MHz [32], the limiting factor for scanning speeds is in the sensor used, with standard CMOS sensors showing frame rates usually between 30 fps up to 1000 fps, which bounds the scanning speed to this same number of pixels per second. So far, the prototypes presented are slower compared to other optical-scanning techniques, such as scanning confocal microscopy, which presents a scanning speed of hundreds of kSamples/s, acquiring an image in few seconds [33]. However, the image-acquisition speed was not considered, since the aim was to validate the proposed method and study the resolving power and how to improve the FOV.

## Data Availability

Data sharing not applicable.
